# Distribution and Characteristics of Oral Pathogens According to Blood Glucose Levels in South Korean Health Examinees

**DOI:** 10.3390/ijms26062638

**Published:** 2025-03-14

**Authors:** Yong Jun Choi, Jooheon Park, Myung Geun Shin, Bong-Kwang Jung, Hyejoo Shin, Seon Cho, Han-Ik Cho, Eun-Hee Nah

**Affiliations:** 1 Department of Laboratory Medicine, Chonnam National University Hwasun Hospital, Hwasun 58128, Republic of Korea; azarsis@hanmail.net (Y.J.C.); mgshin@chonnam.ac.kr (M.G.S.); 2 MEDIcheck Research Institute, Korea Association of Health Promotion, Seoul 07572, Republic of Korea; mulddang@snu.ac.kr (B.-K.J.); hyejoo0422@naver.com (H.S.); dduddi3755@hanmail.net (S.C.); 3 MEDIcheck LAB, Korea Association of Health Promotion, Seoul 07572, Republic of Korea; hanik@snu.ac.kr; 4 Department of Laboratory Medicine, Chonnam National University Hospital, Gwangju 61469, Republic of Korea

**Keywords:** oral pathogen, periodontitis, dental caries, multiplex real-time polymerase chain reaction, diabetes mellitus

## Abstract

The distribution of oral pathogens is influenced by genetic background, diet, socioeconomic status, and racial factors. This study aimed to assess the distribution and characteristics of oral pathogens based on blood glucose levels in a South Korean population. This cross-sectional, retrospective study included subjects from 17 health promotion centers in 13 South Korean cities between November 2021 and December 2022. Real-time multiplex PCR was used to detect 10 periodontitis-related pathogens, 6 dental caries-related pathogens, and 1 dental caries-protective bacterium. The most prevalent periodontitis-related pathogens were *Parvimonas micra* (97.6%), *Porphyromonas endodontalis* (96.8%), and *Treponema socranskii* (95.0%). Among dental caries-related pathogens, *Streptococcus sanguinis* and *Veillonella parvula* were found in all subjects. The prevalence of periodontitis-related pathogens was higher in males, while pathogens related to periodontitis and dental caries were more prevalent in older individuals. In the diabetes group, *Aggregatibacter actinomycetemcomitans*, red and orange complexes, and *Streptococcus mutans* were more prevalent. The relative amount of *S*. *sanguinis* was lower, while *V*. *parvula* was higher in individuals with diabetes mellitus. The prevalence and composition of oral pathogens vary by sex, age, and blood glucose levels. Diabetic individuals showed a pathogenic community structure linked to increased risks of periodontitis and dental caries.

## 1. Introduction

The oral microbiome is one of the most diverse and dynamic ecosystems of the human body, containing more than 1000 bacterial species [1]. These microorganisms normally coexist symbiotically with their host. However, behavioral factors such as poor oral hygiene, debilitated immune system, genetics, medication, and certain diseases can lead to a dysbiotic oral ecosystem [2]. This imbalance is associated with pathogenic microorganism overgrowth, which can increase susceptibility to oral illnesses such as periodontal disease or dental caries [3]. The human microbiome varies significantly with the genetic background, diet, socioeconomic status, and living environment of the population [4]. Several studies have found that the human microbiome varies markedly between geographical locations and ethnicities [5,6]. However, few studies have been conducted on the distribution and composition of oral pathogens in community-dwelling South Koreans.

The oral microbiome has also been associated with systemic diseases such as diabetes mellitus (DM) and oral diseases such as periodontitis [7,8,9]. One study found that patients with DM had lower biological and phylogenetic oral microbiome diversity compared to non-diabetics [10]. Although there are no phenotypic features unique to periodontitis in patients with DM, DM increases periodontitis risk [11]. Nonetheless, the effects of DM on the oral microbiome remain controversial.

Technological advances have led to the development of many methods for detecting and quantifying the oral microbiome, such as the real-time PCR assay, which allows accurate quantification with higher sensitivity, specificity, simplicity, and rapidity [12,13]. In particular, the multiplex real-time PCR assay can help in assessing the relative abundance of several bacterial species that are relevant to oral health.

This study aimed to determine the distribution and composition of oral pathogens and their characteristics according to blood glucose levels using a multiplex real-time PCR assay in South Korean health checkup examinees.

## 2. Results

This study included 3045 subjects aged 57.0 ± 9.7 years (range = 18–97 years), and 56.2% were male (Table 1).

### 2.1. Prevalence of Periodontitis and Dental Caries Pathogens According to Sex and Age

**Figure 1:**
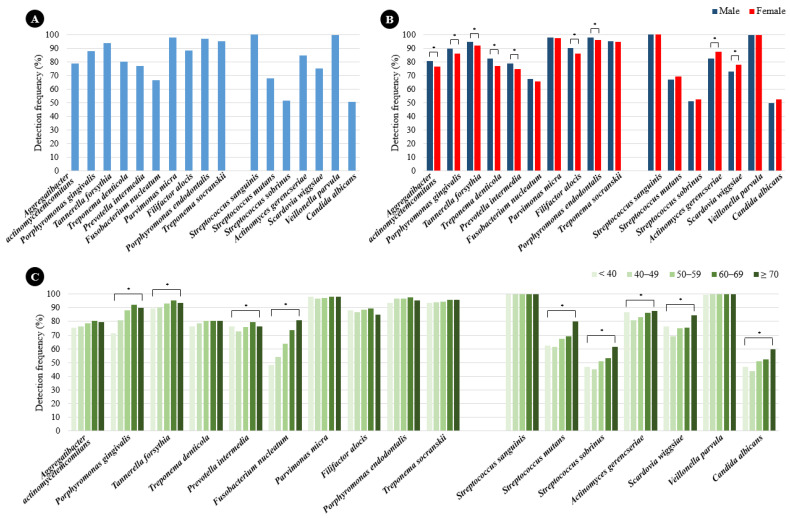
(**A**) Prevalence of oral pathogens among 3045 South Korean individuals undergoing health checkups, as determined by qPCR analysis of subgingival and dental plaque samples. (**B**) Bacterial detection frequency in males and females. (**C**) Bacterial detection frequency according to age. * *p* < 0.05 as calculated using the Chi-square test.

The prevalence rates of periodontitis and dental caries pathogens among the study subjects are shown in Figure 1A. *Parvimonas micra* (97.6%), *Porphyromonas endodontalis* (96.8%), and *Treponema socranskii* (95.0%) were highly prevalent, and *Fusobacterium nucleatum* (66.6%) was the least prevalent. The prevalence rates of periodontitis pathogens were significantly higher in males than in females except for *F*. *nucleatum*, *P*. *micra*, and *T*. *socranskii* (Figure 1B). *Porphyromonas gingivalis*, *Tannerella forsythia*, *Prevotella intermedia*, and *F*. *nucleatum* were more prevalent in older subjects (Figure 1C). The prevalence rates of dental caries pathogens among the study subjects are shown in Figure 1A. *Streptococcus sanguinis* and *Veillonella parvula* were detected in all subjects, while *Candida albicans* (50.8%) was the least prevalent. The prevalence rates of *Actinomyces gerencseriae* and *Scardovia wiggsiae* were significantly higher in females than in males (Figure 1B). The prevalence rates of all dental caries pathogens, with the exceptions of *S*. *sanguinis* and *V*. *parvula*, increased with age (Figure 1C).

### 2.2. Quantification of Periodontitis and Dental Caries Pathogens According to Sex and Age

**Figure 2:**
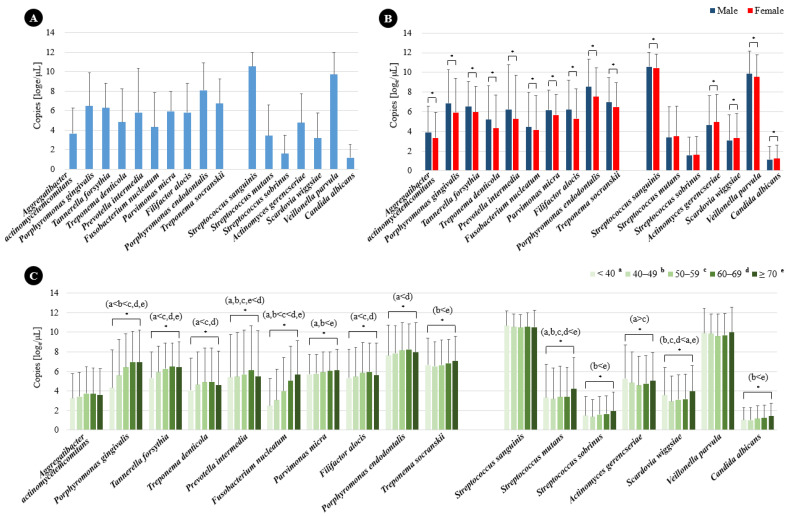
Bacterial content (DNA copy number) of periodontitis and dental caries, as determined by qPCR analysis of subgingival and dental plaque samples: (**A**) Total bacterial content; (**B**) bacterial content by sex; (**C**) bacterial content by age. Data are mean and standard deviation values. * *p* < 0.05 as calculated using *t*-test and ANOVA with the post-hoc Scheffe’s method.

The DNA copy numbers of the periodontitis and dental caries pathogens among the study subjects are shown in Figure 2A. *P*. *endodontalis* emerged as the most abundant periodontitis pathogen, while *Aggregatibacter actinomycetemcomitans* was identified as the least abundant. In terms of dental caries pathogens, *S*. *sanguinis* was the most abundant, whereas *C*. *albicans* was the least. A significant disparity was observed in the total abundance of periodontitis pathogens, which was notably higher in males compared to females (*p* < 0.05) (Figure 2B). Additionally, this abundance, excluding *A. actinomycetemcomitans*, also showed an increase with advancing age. Interestingly, *A. gerencseriae* and *S*. *wiggsiae* were abundant in individuals under 40 years old compared to other age groups (*p* < 0.05) (Figure 2C). Furthermore, *S*. *sanguinis* and *V*. *parvula* were found to be significantly more abundant in males, in contrast to *A*. *gerencseriae*, *S*. *wiggsiae*, and *C*. *albicans*, which were significantly more abundant in females (*p* < 0.05) (Figure 2B). The quantities of all dental caries pathogens, with the exception of *S*. *sanguinis* and *V*. *parvula*, were observed to increase with age (Figure 2C).

### 2.3. Prevalence Rates and Percentage Contents of Periodontitis and Dental Caries Pathogens According to Blood Glucose Levels

**Figure 3:**
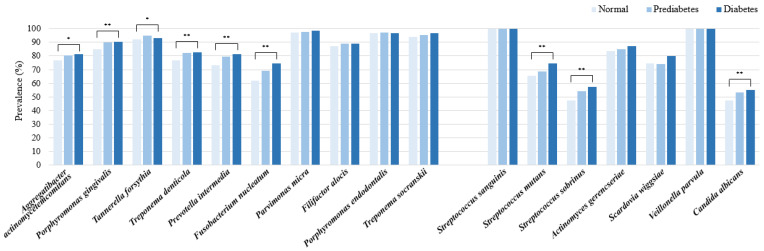
Prevalence of oral pathogens according to blood glucose levels. * *p* < 0.05; ** *p* < 0.01 as calculated using Chi-square test.

**Figure 4:**
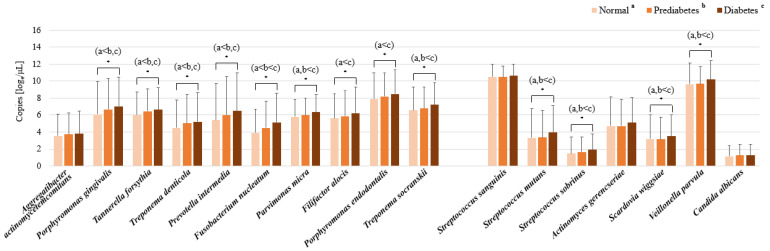
Bacterial content (DNA copy number) of periodontitis and dental caries by blood glucose status, as determined by qPCR analysis of subgingival and dental plaque samples. Data are mean and standard deviation values. * *p* < 0.05 as calculated using ANOVA with the post-hoc Scheffe’s method.

*P*. *micra*, *P*. *endodontalis*, *T*. *socranskii*, and *T*. *forsythia* emerged as the predominant periodontitis pathogens in both the normoglycemic and diabetic cohorts. Notably, *A*. *actinomycetemcomitans*, along with the red and orange complexes, exhibited a higher prevalence within the diabetes group (*p* < 0.05). In terms of dental caries pathogens, *S*. *sanguinis*, *V*. *parvula*, and *A*. *gerencseriae* were identified as the most common across all three blood glucose categories. However, *Streptococcus mutans*, *Streptococcus sobrinus*, and *C*. *albicans* were found to be more prevalent in the diabetes group (*p* < 0.005) (Figure 3). A significant increase in the abundance of all periodontitis pathogens was observed in the diabetes group compared to the normoglycemic group, with the exception of *A*. *actinomycetemcomitans*. Among the dental caries pathogens, *S*. *mutans*, *S*. *sobrinus*, *S*. *wiggsiae*, and *V*. *parvula* were significantly more abundant in the diabetes group than in the normoglycemic group (*p* < 0.001) (Figure 4). In the context of relative abundance, *P*. *micra* exhibited a markedly lower percentage content in the prediabetes group compared to the normoglycemic group. Concerning pathogens associated with dental caries, the relative abundance of *S*. *sanguinis* was significantly diminished, whereas *V*. *parvula* presented a notably increased percentage content in the diabetes group compared to the normoglycemic group (*p* < 0.001) (Table 2).

## 3. Discussion

This study demonstrated the distribution of periodontitis and dental caries pathogens and their characteristics according to blood glucose levels in a South Korean community-dwelling cohort with a wide age range. Periodontitis and dental caries pathogens were found in the oral cavities of apparently healthy dental examinees. The prevalence rates and quantity of most pathogens differed between sex and age groups. The prevalence rates of red and orange complexes were higher in DM than in normoglycemia. Moreover, the relative abundance of some pathogens varied with the blood glucose level, which suggests that oral pathogens have a dysbiotic nature in DM.

Some previous studies also found periodontitis pathogens in both patients with periodontal disease and healthy subjects [14,15,16]. *P*. *micra* (97.6%), *P*. *endodontalis* (96.8%), and *T*. *socranskii* (95.0%) were the most prevalent among ten periodontitis pathogens in our study, and *F*. *nucleatum* (66.6%) was the least prevalent. These results were inconsistent with those of a previous study [14], in which *F*. *nucleatum* was not only the most prevalent overall, but also the most prevalent in healthy South Korean young adults. The earlier research employed a distinct PCR kit that excluded the strains of *P*. *micra*, *P*. *endodontalis*, and *T*. *socranskii*, which were identified as the most prevalent in the current study. Additionally, variations in the age demographics of the participants in the previous study may further account for these discrepancies.

*A*. *actinomycetemcomitans* is recognized as a pathogen associated with aggressive periodontitis in both young adolescents and adults [17]. In our study, although *A*. *actinomycetemcomitans* was frequently identified, its amount was the lowest among the periodontitis pathogens examined, which aligns with the fact that the subjects were healthy individuals. It is noteworthy that potential pathogenic bacteria were regarded as part of the commensal oral microflora. The bacterial load at periodontal disease sites is a critical factor influencing the progression of the disease [18,19]. Furthermore, the red-complex pathogens, specifically *P*. *gingivalis*, *T*. *forsythia*, and *Treponema denticola* were detected at rates of 87.7%, 93.3%, and 79.8%, respectively, in our investigation. These results are in agreement with findings from a Swedish study, which reported the presence of *P*. *gingivalis*, *T*. *forsythia*, and *T*. *denticola* in 82%, 82%, and 94% of healthy Swedish participants, respectively [20]. Such findings indicate that oral pathogens significantly contribute to the onset and progression of oral diseases. Recent perspectives suggest that oral diseases are not attributable to a single pathogen, but rather to microbial dysbiosis [21,22,23]. Disruption of the subgingival plaque can impair the immune response within the gum tissue, leading to further imbalances in host–bacteria interactions and potentially resulting in severe dysbiosis of the microbiota [24].

The present study found that periodontitis and dental caries pathogens varied with sex. The prevalence rates of red and orange complexes and the DNA copy numbers of periodontitis pathogens were significantly higher in males in the present study. Some other studies have also found sex differences in oral pathogens such as periodontitis and dental caries pathogens [14,25]. Lira-Junior et al. [25] reported that males had more oral pathogens in the saliva, which was consistent with the results of the present study. They speculated that this could be due to males having a slightly worse periodontal condition than females. On the other hand, we also found that the prevalence of the dental caries pathogens, *A*. *gerencseriae* and *S*. *wiggsiae*, were significantly higher in females than in males.

The amount of most periodontitis and dental caries pathogens increased with age in the present study. This finding was consistent with the previous finding of aging being associated with increased counts of several bacteria, including periodontitis pathogens [25]. That study suggested that aging is associated with increased levels of inflammatory biomarkers in saliva along with an altered microbial profile. Age-related alterations in salivary microbiota have been observed in previous studies [26,27]. Interestingly, *A*. *gerencseriae* and *S*. *wiggsiae* were abundant in individuals under 40 years old compared to other age groups within our study. It has been proposed that additional research is warranted to explore these variations during early and late adulthood.

Individuals with periodontitis are more potentially to develop DM since periodontal diseases may contribute to the body’s overall inflammatory burden [28]. A bidirectional relationship between periodontal diseases and DM has been suggested [11]. A notable rise in the abundance of various periodontitis and dental caries pathogens was identified in the diabetes cohort when compared to the normoglycemic cohort in our research. Specifically, *A*. *actinomycetemcomitans*, along with the red and orange complexes, exhibited greater prevalence within the diabetes group. In the context of relative abundance, *P*. *micra* exhibited a markedly lower percentage content in the prediabetes group compared to the normoglycemic group. The percentage content of *A*. *actinomycetemcomitans* in individuals with diabetes was lower than that found in those with normal blood glucose levels; however, this difference did not achieve statistical significance in the present study. Additionally, the proportion of dental caries-protective bacteria *S*. *sanguinis* was significantly diminished, whereas the percentage content of *V*. *parvula* was markedly elevated in the diabetes group relative to the normoglycemic group in our study. Recent studies [29,30,31] using next-generation sequencing technology indicated that DM could alter the biodiversity and composition of the oral microbiome, especially subgingival microbiomes. A study found that a higher abundance of *Actinobacteria* was associated with lower diabetes risk, as they were less abundant among diabetic subjects compared to normal controls [30]. The oral microbial communities are normally stable and symbiotic in healthy individuals. Interruption of this balance by external factors such as DM results in the microbial communities dramatically shifting from a symbiotic to a dysbiotic state, which is likely to induce periodontal disease and dental caries [32,33,34]. In a clinical study [35], researchers identified notable changes in the expression levels of *F*. *nucleatum* and *T*. *forsythia* in patients with DM compared to healthy control subjects. These expression levels exhibited a positive correlation with both fasting blood glucose and glycated hemoglobin concentrations. Furthermore, the study clarified the molecular mechanism by which *F*. *nucleatum* triggers cytokine release through the toll-like receptor 2 signaling pathway in human gingival epithelial cells, as well as the impact of these cytokines on insulin resistance pathways within liver cells. Although the most apparent feature of dysbiosis is the overgrowth of pathogenic microorganisms such as the red complex, they are not the sole cause of periodontitis, and instead synergize to initiate the disease [36].

This study was subject to some limitations. First, it had a cross-sectional study and could, therefore, not confirm the presence of any causal relationship. Large longitudinal studies are needed to assess the relationship between DM and oral pathogens. Second, this study did not consider the factors that tend to affect oral pathogens, such as diet, lifestyle, and medication use. This study did not explore the relationship between the species load, which varied significantly among the three groups, and glucose levels. Furthermore, it did not encompass other biochemical parameters within the scope of this study. Future investigations are necessary to address these limitations. Third, the oral microbiome could not be surveyed more comprehensively due to the relatively small number of different bacteria that were analyzed using multiplex real-time PCR in our study. And this study does not validate the conclusions with further sequencing results. Next-generation sequencing methodologies would be more helpful for completely characterizing the oral microbiome. However, these technologies are currently expensive and far from accessible for the routine analysis of samples in most clinical laboratories. The authors express a desire to confirm the outcomes of this study through sequencing methods in forthcoming research.

In conclusion, this study found that sex, age, and blood glucose levels were associated with changes in the distribution and relative composition of oral pathogens in clinically healthy periodontium and teeth. The oral microbiome in patients with DM has a disease-associated community framework, predisposing those patients to developing periodontal disease and dental caries. Evaluating the oral microbiome would be valuable for preventing the development of periodontal disease and dental caries in patients with DM, even among those in a clinically healthy dental state.

## 4. Materials and Methods

### 4.1. Study Subjects

This cross-sectional, retrospective study consecutively selected subjects who underwent dental examinations, including tests for periodontitis and dental caries pathogens, at 17 health-promotion centers in 13 South Korean cities between November 2021 and December 2022. The Korea Association of Health Promotion runs a health checkup program that includes dental examinations provided either by the Korean National Health Insurance Service or are paid for privately. The inclusion criteria for subjects were as follows: age equal to or older than 18 years old, no history of cancer, and people subjected to the oral microbiome tests. Subjects who did not undergo oral microbiome tests or who had missing values in the biochemical tests, which are prerequisites for the assessment, were excluded. Blood glucose levels were classified in 3 categories: (i) normal (fasting blood glucose < 100 mg/dL), (ii) prediabetes (100 mg/dL ≤ fasting blood glucose < 126 mg/dL), and (iii) diabetes (fasting blood glucose ≥ 126 mg/dL).

### 4.2. Laboratory Measurements

Blood samples were collected from the antecubital vein of each subject while in a sitting position after fasting for >8 h. Blood samples in EDTA tubes were stored at room temperature and analyzed by a technician within 2 h of collection. Complete blood count parameters, including hemoglobin level, were measured using the Sysmex XE-2100D analyzer (Sysmex, Kobe, Japan). Biochemical measurements, including those of serum glucose, liver function tests, and lipid profiles, were made using the Hitachi 7600 analyzer (Hitach, Tokyo, Japan).

### 4.3. Sample Collection and DNA Extraction

Subgingival and dental plaque samples were collected from dental health examinees using a collection and transport system (Noble Bio, Whasung, Republic of Korea) and stored at 4 °C until used. For swab pretreatment, a dry swab was suspended in 200 μL of bacterial lysis buffer and 20 μL of proteinase K was added to the swabs in the transport medium. The liquid sample was then incubated at 65 °C for 10 min, followed by 10 min of incubation at 95 °C. The bacterial genomic DNA was extracted using the MagNA Pure 96 DNA and Viral NA Small Volume Kit (Roche Diagnostics, Manheim, Germany) according to the manufacturer’s instructions.

### 4.4. Multiplex Real-Time PCR

The real-time PCR was performed using the PowerCheck Periodontitis Pathogens Multiplex Real-time PCR kit and PowerCheck Dental Caries Pathogens Multiplex Real-time PCR kit (KogeneBiotech, Seoul, Republic of Korea). The former kit was developed for detecting 10 periodontitis-related pathogens including *A*. *actinomycetemcomitans*, red complex [*P*. *gingivalis*, *T*. *forsythia*, *T*. *denticola*], orange complex [*P*. *intermedia*, *F*. *nucleatum*, *P*. *micra*], and others [*Filifactor alocis*, *P*. *endodontalis*, *T*. *socranskii*], while the latter was developed for detecting 6 dental caries-related pathogens and 1 dental caries-protective bacteria. Oral bacteria and their target genes are listed in Table 3. Amplifications were performed through preheating and initial melting at 50 °C and 95 °C for 2 and 10 min, respectively, followed by 45 cycles of denaturing at 95 °C. Heating and cooling were carried out for 15 s, and extension was performed for 1 min at 60 °C. Standard curves for each organism were plotted in each primer–probe set using the cycle threshold values obtained by amplifying successive 10-fold dilutions of the known copies of the bacteria. Real-time PCR was performed and analyzed using the CFXOpus-384 Real-Time PCR Detection System (Bio-Rad, Hercules, CA, USA).

### 4.5. Statistical Analysis

Statistical analyses were performed using SAS software (version 9.4, SAS Institute, Cary, NC, USA). Data are expressed as mean ± standard deviation (SD) and frequency (percentage) values. The bacterial detection frequency in the study subjects was calculated as a percentage. The DNA copy numbers of the oral pathogens were displayed as a bar graph with the means and SDs of the detected pathogens. Sex differences in the prevalence rates and DNA copy numbers of the pathogens were analyzed using Chi-square and Student’s *t*-tests, respectively. These differences according to age were analyzed using the Chi-square test and ANOVA with the post-hoc Scheffe’s method, respectively. The percentage content of each pathogen was used to compare the relative abundance of bacteria in total periodontitis or dental caries pathogens. The percentage content of each pathogen refers to the ratio of the DNA quantity of a particular bacterium in relation to the overall total of periodontitis or dental caries pathogens. This calculation is achieved by dividing the DNA quantity of each individual pathogen by the total DNA amount of all periodontitis or dental caries pathogens, followed by multiplying the result by 100. The differences in prevalence rates and percentage contents of oral pathogens among blood glucose groups were analyzed using the Chi-square test and ANOVA with the post-hoc Scheffe’s method, respectively. Significance was accepted at a level of *p* < 0.05.

## Figures and Tables

**Table 1:** Demographic and clinical characteristics of the study subjects.

Characteristic	Total (n = 3045)	Normal ^a^ (n = 1352)	Prediabetes ^b^ (n = 1305)	Diabetes ^c^ (n = 388)	*p* *	Multiple Comparisons
Age, years	57 ± 9.7	55.5 ± 10.3	57.9 ± 9.2	59.3 ± 8.6	<0.001	a < b < c
Sex, male	1711 (56.2)	656 (48.5)	771 (59.1)	284 (73.2)	<0.001	a < b < c
Blood glucose, mg/dL	107.2 ± 24.4	91.6 ± 6.2	108.8 ± 6.7	156 ± 33.9	<0.001	a < b < c
AST, U/L	34 ± 21.6	31.9 ± 17.5	34.5 ± 21.5	39.9 ± 31.2	<0.001	a < b < c
ALT, U/L	33 ± 51.3	28.7 ± 21.8	33.4 ± 25.2	46.8 ± 129	<0.001	a, b < c
γ-GTP, U/L	44.9 ± 69.9	36.5 ± 63.4	48.8 ± 76.3	61.3 ± 64.5	<0.001	a < b < c
Total cholesterol, mg/dL	207 ± 48.5	210.8 ± 40.6	208.1 ± 44.2	189.7 ± 76.3	<0.001	a, b > c
Triglycerides, mg/dL	125.9 ± 99.8	105 ± 74.4	134.2 ± 86.8	170.4 ± 172.8	<0.001	a < b < c
HDL-cholesterol, mg/dL	55.3 ± 14.1	58 ± 15	54.2 ± 13.1	49.7 ± 11.8	<0.001	a > b > c
LDL-cholesterol, mg/dL	123.1 ± 40	127.2 ± 38.1	124.1 ± 40.1	105 ± 41.5	<0.001	a, b > c
Hemoglobin, g/dL	14.6 ± 1.5	14.4 ± 1.5	14.7 ± 1.4	15 ± 1.5	<0.001	a < b < c

Blood glucose levels were classified in 3 categories: (i) normal (fasting blood glucose < 100 mg/dL), (ii) prediabetes (100 mg/dL ≤ fasting blood glucose < 126 mg/dL), and (iii) diabetes (fasting blood glucose ≥ 126 mg/dL). Data are mean ± standard deviation or n (%) values. * Statistical analysis was performed using ANOVA with the post-hoc Scheffe’s method. Abbreviations: AST, aspartate aminotransferase; ALT, alanine aminotransferase; γ-GTP, gamma glutamyl transpeptidase; HDL, high-density lipoprotein; LDL, low-density lipoprotein.

**Table 2:** Percentage content of each pathogen in oral pathogens according to blood glucose levels.

Bacterial Strain	Total (*n* = 3041)	Normal ^a^ (*n* = 1348)	Prediabetes ^b^ (*n* = 1305)	Diabetes ^c^ (*n* = 388)	*p* *
**Periodontitis pathogens**					
*Aggregatibacter* *actinomycetemcomitans*	1.5%	1.9%	1.3%	1.0%	0.054
*Porphyromonas gingivalis*	12.1%	11.6%	12.6%	12.1%	0.227
*Tannerella forsythia*	5.7%	5.7%	5.8%	5.6%	0.922
*Treponema denticola*	4.0%	3.7%	4.3%	4.0%	0.053
*Prevotella intermedia*	23.0%	21.7%	23.8%	24.8%	0.058
*Fusobacterium nucleatum*	6.2%	5.9%	6.7%	5.8%	0.168
*Parvimonas micra*	4.4%	4.9%	4.1%	4.0%	0.023
*Filifactor alocis*	4.7%	4.7%	4.6%	4.8%	0.964
*Porphyromonas endodontalis*	28.6%	29.6%	27.6%	28.7%	0.077
*Treponema socranskii*	9.8%	10.3%	9.2%	9.2%	0.114
**Dental caries pathogens**					
*Streptococcus sanguinis*	59.7%	61.1%	59.8%	54.2%	<0.001
*Streptococcus mutans*	1.6%	1.6%	1.6%	2.1%	0.339
*Streptococcus sobrinus*	0.2%	0.2%	0.2%	0.2%	0.974
*Actinomyces gerencseriae*	1.8%	1.7%	1.9%	1.9%	0.718
*Scardovia wiggsiae*	0.7%	0.7%	0.6%	0.7%	0.558
*Veillonella parvula*	35.9%	34.6%	35.8%	40.8%	<0.001
*Candida albicans*	0.1%	0.1%	0.1%	0.1%	0.735

* The results of multiple comparisons: *Parvimonas micra* a > b, c; *Streptococcus sanguinis* a, b > c; *Veil-lonella parvula* a, b < c.

**Table 3:** Target genes of oral bacteria contained in the PowerCheck Periodontitis Pathogens Multiplex Real-time PCR kit and PowerCheck Dental Caries Pathogens Multiplex Real-time PCR kit.

Bacterial Strain	Target Gene
**Periodontitis pathogens**	
*Aggregatibacter actinomycetemcomitans*	*fts I*
*Prohormonal gingivalis*	*waaA*
*Tannerella forsythia*	*fts Z*
*Treponema denticola*	*fts K*
*Prevotella intermedia*	*piACP*
*Fusobacterium nucleatum*	*rpoB*
*Parvimonas micra*	*fus A*
*Filifactor alocis*	*gyr B*
*Porphyromonas endodontalis*	16S
*Treponema socranskii*	16S
**Dental caries pathogens**	
*Streptococcus sanguinis*	*tuf*
*Streptococcus mutans*	*gtf B*
*Streptococcus sobrinus*	*gtf I*
*Actinomyces gerencseriae*	16S
*Scardovia wiggsiae*	16S
*Veillonella parvula*	*rpoB*
*Candida albicans*	*SAP3*

## Data Availability

The raw data supporting the conclusions of this article will be made available by the authors on request.
